# Terrestrial Contributions to the Aquatic Food Web in the Middle Yangtze River

**DOI:** 10.1371/journal.pone.0102473

**Published:** 2014-07-21

**Authors:** Jianzhu Wang, Binhe Gu, Jianhui Huang, Xingguo Han, Guanghui Lin, Fawen Zheng, Yuncong Li

**Affiliations:** 1 Collaborative Innovation Center for Geo-hazards and Eco-environment in Three Gorges Area, Hubei Province, The Three Gorges University, Yichang, China; 2 Soil and Water Science Department, University of Florida, Gainesville, Florida, United States of America; 3 Institute of Botany, the Chinese Academy of Sciences, Beijing, China; 4 Institute of Applied Ecology, the Chinese Academy of Sciences, Shenyang, China; 5 Center for Earth System Science, Tsinghua University, Beijing, China; 6 Nanjing Research Institute of Hydrology and Water Resources, Nanjing, China; 7 Soil and Water Science Department, Tropical Research and Education Center, IFAS, University of Florida, Homestead, Florida, United States of America; University of Shiga Prefecture, Japan

## Abstract

Understanding the carbon sources supporting aquatic consumers in large rivers is essential for the protection of ecological integrity and for wildlife management. The relative importance of terrestrial and algal carbon to the aquatic food webs is still under intensive debate. The Yangtze River is the largest river in China and the third longest river in the world. The completion of the Three Gorges Dam (TGD) in 2003 has significantly altered the hydrological regime of the middle Yangtze River, but its immediate impact on carbon sources supporting the river food web is unknown. In this study, potential production sources from riparian and the main river channel, and selected aquatic consumers (invertebrates and fish) at an upstream constricted-channel site (Luoqi), a midstream estuarine site (Huanghua) and a near dam limnetic site (Maoping) of the TGD were collected for stable isotope (δ^13^C and δ^15^N) and IsoSource analyses. Model estimates indicated that terrestrial plants were the dominant carbon sources supporting the consumer taxa at the three study sites. Algal production appeared to play a supplemental role in supporting consumer production. The contribution from C_4_ plants was more important than that of C_3_ plants at the upstream site while C_3_ plants were the more important carbon source to the consumers at the two impacted sites (Huanghua and Maoping), particularly at the midstream site. There was no trend of increase in the contribution of autochthonous production from the upstream to the downstream sites as the flow rate decreased dramatically along the main river channel due to the construction of TGD. Our findings, along with recent studies in rivers and lakes, are contradictory to studies that demonstrate the importance of algal carbon in the aquatic food web. Differences in system geomorphology, hydrology, habitat heterogeneity, and land use may account for these contradictory findings reported in various studies.

## Introduction

Understanding the relative importance of terrestrial and aquatic sources of the carbon that supports food webs in large rivers is essential for floodplain management and for wildlife conservation. Three conceptual models have been proposed to examine the contribution of various carbon sources to the lotic food webs. The river continuum concept (RCC) proposes that the major source of organic matter supporting the large river food webs originates from terrestrial plants from the headwater and mid-streams, while in-stream primary production is limited by turbidity and light attenuation associated with depth [41], [47]. The flood pulse concept (FPC) emphasizes the importance of lateral river floodplain exchanges and proposes that river food webs are more dependent on production derived from the floodplain than on organic matter transported from upstream [26]. The riverine productivity model (RPM) [44], however, highlights the importance of local in-stream production (phytoplankton, benthic algae, and other aquatic plants). Thorp et al [46] examined these three food web theories within a floodplain and a constricted-channel reach of the Ohio River. They argued that the RPM is the better model for channel-constricted regions in the Ohio River, but could not be generally applied until similar food web studies are conducted in different types of rivers throughout temperate and tropical latitudes. The RPM and the importance of in-stream autotrophs were further verified in a study on the Upper Mississippi River using stable isotope analysis [46]. They argued that the nutritionally poor, and sometime recalcitrant terrestrial organic matter cannot be the major source of carbon to the river food web. Other studies also identify algal carbon as the dominant energy source, fueling the river and lacustrine food webs around the world [3], [4], [22], [43], [45].

By contrast, there is also strong evidence that terrestrially-derived organic matter is an essential source of carbon to the receiving waters and may dominate aquatic consumer production in streams [38.48] and rivers [23], [25], [57]. About 70% to 90% of all primary production eventually enters the detritus food web [36] and exceeds autochthonous production [21]. Wallace et al [48] conducted a large-scale, three-year exclusion of terrestrial leaf litter input to a forest stream. The exclusion of leaf litter led to the decline of abundance, biomass or both of the invertebrate taxa compared with those in a reference stream. This study demonstrated the importance of riparian detritus as an essential carbon source to the stream food web. Recently, whole lake ^13^C enrichment revealed that terrestrial carbon played an important role in zooplankton and fish production [7], [33]. Zeug and Winemiller [57] collected various production sources and aquatic consumers from the Brazos River for stable isotope and IsoSource mixing model analysis and identified terrestrial C_3_ plants as the dominant carbon source. Doucett et al [12] and Cole et al [8] found large differences in the natural abundance of hydrogen stable isotopes between terrestrial plants and aquatic primary producers and indicated that terrestrial carbon was a significant contributor to zooplankton in small lakes and to benthic invertebrates and fish in streams and rivers. Considering the existence of contradictory findings on the relative contribution of terrestrial and aquatic carbon sources to the river food web, further work is required to gain greater insight into the energy flow and nutrient cycling pathways in large rivers under vastly different morphological, biological and ecological conditions.

**Figure 1:**
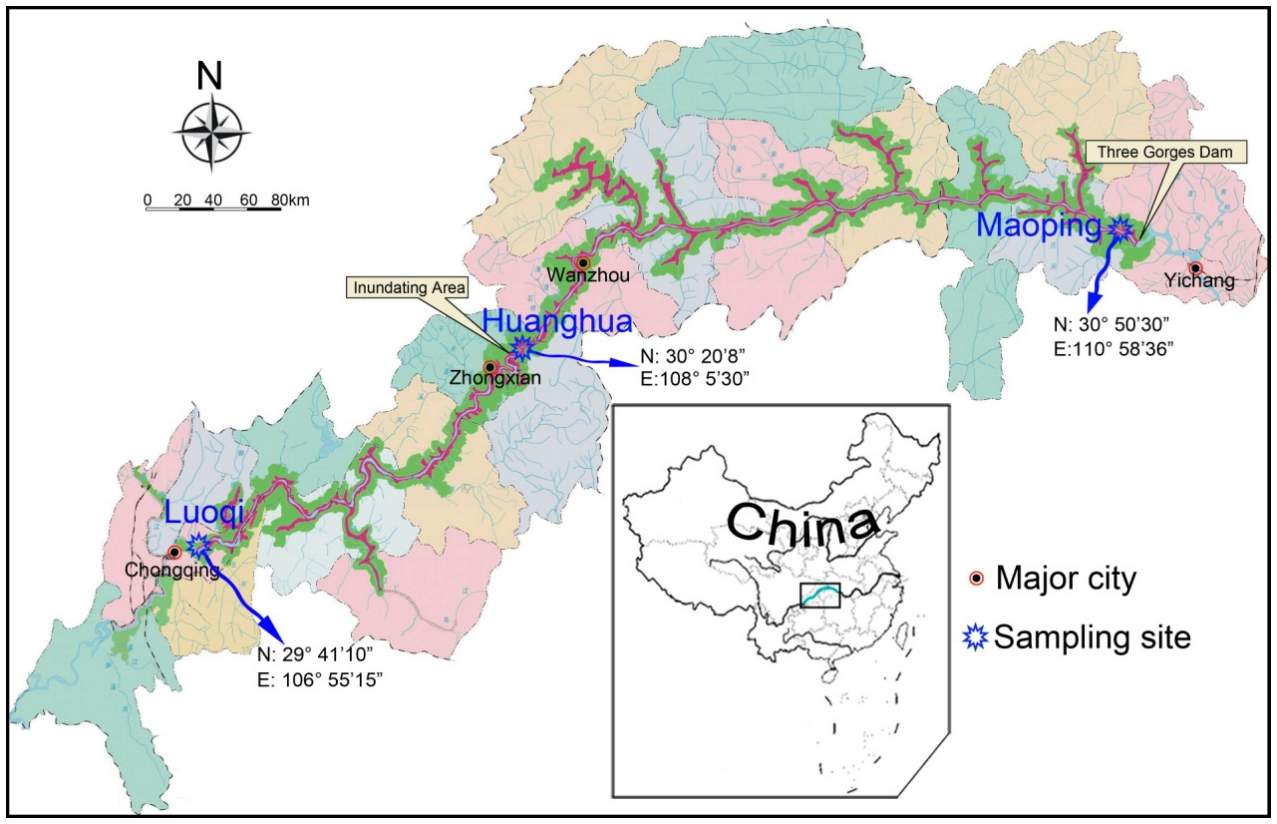
Map of the Three Gorges Dam area showing the three study sites and major cities.

**Figure 2:**
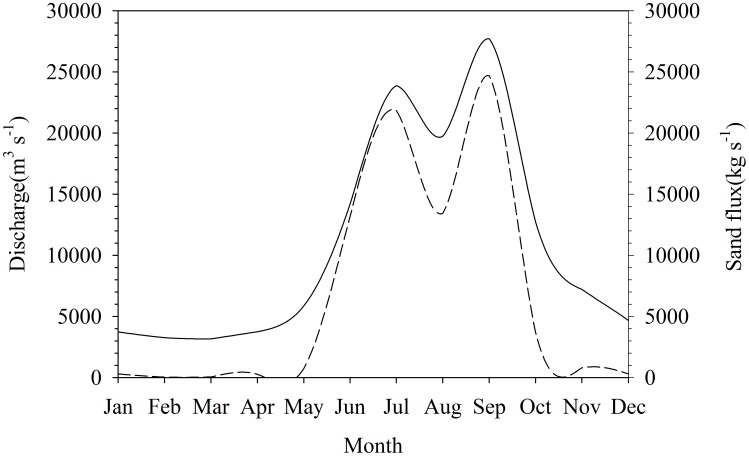
Monthly water discharge (solid line) and sand flux (dash line) at the Luoqi site on the upper Yangtze River in 2004.

The Yangtze River is the largest river in China and the third longest river in the world, with a length of 6,300 km and a drainage basin of 1,808,500 km^2^ (Fig. 1). Tremendous periodic flood events occur in the summer with a dry period during the winter and spring (Fig. 2). The average hydrological discharge is 30,166 m^3^ s^−1^ with a maximum of 110,000 m^3^ s^−1^ and a minimum of 2,000 m^3^ s^−1^ under natural condition. The Yangtze River has a highly diverse freshwater fish community, with 361 fish species from 29 families and 131 genera, accounting for 36% of all freshwater fish species in China [17], [54]. The Three Gorges Dam (TGD), which is the world's largest dam, has been constructed in the middle Yangtze River in south-central China. As a result, the water depth upstream of the dam had increased by 66 meters since the Three Gorges Reservoir (TGR) began storing water in late 2003. Since damming of rivers is the most dramatic anthropogenic factor affecting freshwater environments [13], [14], [15], construction of the TGD would also have severe environmental consequences [1], [34]. The formerly narrow channel characterized by torrential water flow has been converted to an extensive stagnant water body similar to the limnetic zone of a large lake system, with corresponding increases in the water depth, alternations of the flow rate, dissolved oxygen, light availability and temperature in the TGR [31], [40], [50]. These changes likely would result in an increase in phytoplankton production and a decrease in large vascular plants. On the other hand, large volumes of terrestrial organic matter are discharged into the TGR from the upper Yangtze River and its distributaries during the wet season and will remain in the TGR for a longer time than during the pre-dam period, which also can change the composition of organic matter and further convert the energy sources for the aquatic biome in this region [34], [42].

The combination of a large floodplain area, extreme hydrology and rich fish resources makes the Yangtze River an ideal system with which to examine the three food web models. We hypothesized that terrestrial plants are the dominant source of organic matter to the consumer production in the region of middle Yangtze River where hydrology has not been greatly altered by the TGD. This is due to the extreme flow events, high turbidity and high water depth, which severely limit in-stream primary production. We further hypothesized that the construction of the TGD would increase the contribution of in-stream production to consumer production, which would be especially apparent in the immediate upstream region of the TGD. These hypotheses were tested using stable isotope analyses of the dominant sources of terrestrial and aquatic organic matter, and representative consumer taxa collected at three study sites along the middle Yangteze River, upstream of the TGD during two representative months of the dry and wet periods. The IsoSource mixing model [35] was used to determine the relative contribution of terrestrial C_3_, and C_4_ plants, phytoplankton and benthic algal production.

## Materials and Methods

Ethical approval was given by the ethical committee at the Beijing Institute of Botany, Chinese Academy of Science. In the current study, the use of tissue material from animals killed is a part of routine commercial fishery production. The commercial fishery complies with the local fishery law in Hubei Province. No specific permits were required for the described field studies. The locations studied were not privately-owned or protected in any way and the field studies did not involve endangered or protected species.

### Study sites

The Yangtze River originates in the Geladandong Mountains on the Tibetan Plateau, and follows a sinuous west to east route before empting into the East China Sea at Shanghai (Fig. 1). The main channel of the upper Yangtze River is 1,040 km long and the TGD is located in the lower reaches of the upper Yangtze River. The hydrological cycle in the Yangtze River region includes a dry season from October to May and a wet season from June to early October. This is illustrated by the monthly discharge at Luoqi in 2004 (Fig. 2). The drainage basin of the TGR covers 58,000 km^2^, and encompasses 19 counties in Chongqing and Hubei Province, with three major cities situated on the shorelines of the river channel (Fig. 1). In addition to urban development, agriculture is the other dominant human activity of the region.

The total surface area of the TGR is 1080 km^2^. Our three study sites are located within the TGR from Chongqing to the TGD (Fig. 1). Luoqi (29° 41′10″N, 106° 55′15″E) is about 500 Km upstream of the TGD. It is a typical constricted-channel site with predictable flooding during each summer and its hydrological regime is not affected by the TGD during the study period. Huanghua (30° 20′8″N, 108° 5′30″E) is about 300 Km upstream of the TGD and is affected by the backwater from the dam. Maoping (30° 50′30″N, 110° 58′36″E) is located immediate upstream (<1 km) of the TGD and has displayed a hydrological regime similar to the pelagic zone of a deep-water lake since the completion of the TGD.

### Sample collection and analysis

All samples were collected in two trips to the study sites in September 2004 and May 2005 which represent the wet and dry periods, respectively (Fig. 2). We collected organic matter from eight to ten terrestrial plants (C_3_ and C_4_ plants) in the riparian zone of each site. Leaves from two to three numerically dominant and secondary tree species, seven grasses and agricultural crops were collected. Plant samples were air dried, placed in unsealed envelopes and transported to the laboratory for further processing. The dominant C_3_ plants were *Polygonum hydropiper*, *Conyza canadensis* and *Alternanthera philoxeroides* in Luoqi, *Quercus aliena var. acuteserrata*, *Pterocarya stenoptera*, *Nephrolepis auriculata* and *Ficus tikoua* in in Huanghua and *Pinus massoniana, Quercus aliena var. acuteserrata, Conyza Canadensis and Nephrolepis auriculata* in Maoping. *Cynodondactylon* and *Echinochloa phyllopogon* were dominant C_4_ plants in all sites with *Imperata cylindrica* also abundant in Luoqi.

Epiphytic algae (EA) and filamentous algae (FA, mostly *Spirogyra*) were collected from substrate surface and stored in glass bottles. Samples were rinsed repeatedly in deionized water to remove sediment particles and detritus. Particulate organic matter (POM) was collected from several depths (surface, 5 m, 15 m, 30 m 50 m or deep depth) with a water bottle from two nearshore, two mid-channel and one central station along five cross-transects. Samples from the five stations of each transect were then combined to form a composite sample. In the laboratory, water was passed through coarse (100 µm) sieves and then filtered onto precombusted Whatman GF/C (1.2 µm) glass fiber filters to characterize the coarse POM (CPOM) and fine POM (FPOM; 1.2∼100 µm), respectively. Microscopic examination showed that CPOM was largely consisted of plant detritus while FPOM samples were dominated by planktonic algae.

Four invertebrate taxa were collected. The invertebrates included zooplankton, snails of several species of detritivores/algal grazers, the stream crab (*Sinopotamon yangtsekiense*) and the river shrimp (*Macrobranchium nipponense*) which are detritivores/omnivores. Zooplankton were obtained from vertical hauls of a 100 µm plankton net from each of the five transects at each site. All individuals captured were placed in distilled water at around 4°C for at least 24 h to allow the emptying of their gut contents and then picked under a dissection microscope. All individuals collected from each site of the same transect were combined into a single sample. The snails, stream crabs and river shrimps were hand-collected or captured with fish traps. Shells and gut contents were removed and the individuals rinsed with distilled water. Five individuals of crabs and 5–10 individuals of snails and shrimps were composited into a sample with three replicates for each taxon collected at each site during a sampling event.

A total of 27 species of fish were collected (Table S2, S3 and S4) using various fishing tools including a seine net, fish trap, gillnet or fishing pole. For each fish captured, the total length was measured and three individuals of each species with similar length were chosen for stable isotope analysis. The dorsal white muscle tissue was removed and placed on ice in the field. Six fish species available at all three study sites were selected for the model estimates and comparative analysis. Among the fish species found at all three study sites, the common carp (*Cyprinus carpio*) and the crucian carp (*Carassius auratus auratus*) are omnivores. The bronze gudgeon (*Coreius guichenoti*) is a benthic invertivore while the yellow catfish (*P. fulvidraco*) is an insectivore/invertivore. The Chinese minnow (*Hemiculterella sauvagei*) is a bentho-pelagic feeder relying on the zoobenthos and zooplankton. The Chinese perch (*Siniperca chuatsi*) is a demersal piscivore.

All samples were kept on ice in the field and −20°C in laboratory prior to sample processing. Stable isotope analysis was performed in the Stable Isotope Laboratory for Ecological and Environmental Research at the Research Center for Plant Ecology at the Beijing Institute of Botany, the Chinese Academy of Sciences. Samples were dried at either 105°C for 24 h (plant organic matter) or 60°C for 48 h (consumers) and ground into a fine power using a mortar and pestle, or an agate mill for the vascular plant samples. All laboratory equipment components were cleaned with ethanol, distill water and dried between samples.

The ^13^C/^12^C and ^15^N/^14^N ratios were determined using Thermo Finnigan MAT DELTA^plus^ XP isotope-ratio mass spectrometers. Stable isotope data are expressed as the relative difference ratios of the heavy to light isotopes relative to an internationally accepted reference standard and calculated as:
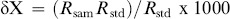
(1)where X is the isotope of interest (^13^C or ^15^N) and; *R*_sam_ and *R*_std_ are the heavy to light isotope ratios of the sample and the standard, respectively. The δX is expressed as the per mil (‰) deviation of that sample from working standards (glycine and cellulose for ^13^C; urea and glycine for ^15^N) and the measurement precision was approximately 0.1 and 0.3‰ for ^13^C/^12^C and ^15^N/^14^N, respectively.

### Data analysis

**Figure 3:**
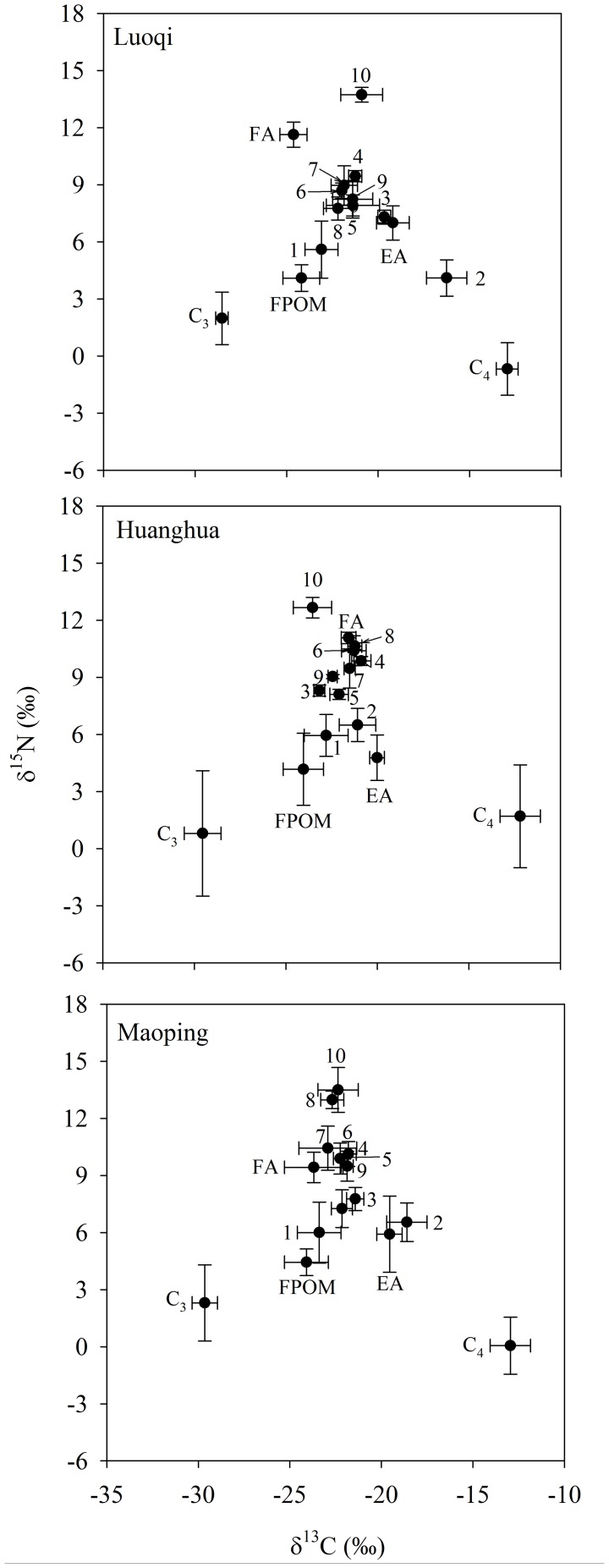
Plots of carbon and nitrogen stable isotope value means and standard deviation error bars (δ^13^C versus δ^15^N) of production sources and consumer assemblages at each study site. Data from the two sampling events were pooled. Symbol codes are as follows: C_3_  =  C_3_ plants, C_4_  =  C_4_ plants, FPOM  =  Fine particulate organic matter, EA  =  epiphytic algae, FA  =  Filamentous algae, 1  =  zooplankton, 2  =  stream crab, 3  =  mixed snails, 4  =  river shrimp, 5  =  common carp, 6  =  crucian carp, 7  =  bronze gudgeon, 8  =  yellow catfish, 9  =  Chinese minnow, 10  =  Chinese perch.

The IsoSource software [35] was used to estimate the contributions of terrestrial and aquatic carbon sources to the consumer assemblage at each study site. Data from the two collection events at each site were pooled prior to the IsoSource analysis. This is because most consumers integrate stable isotope signatures of the dietary resources over time [57] and there was no significant difference in consumer δ^13^C at each site between the dry and wet period ([9] and see Results for more details). There were several potential carbon sources available to the aquatic consumers in the TGD area. These carbon sources included terrestrial C_3_ and C_4_ plants, CPOM and FPOM, EA and FA. The CPOM was largely comprised of terrestrial organic matter shown by the intermediate δ^13^C values (−24.4‰) between those of C_3_ and C_4_ plants at each site (Fig. 3 and Table S1) and was hence excluded from IsoSource model estimates. We used the four invertebrate taxa and six fish species available at all three study sites for the model estimates.

Before isotope data were entered into the IsoSource model, trophic enrichments of δ^13^C and δ^15^N values for each consumer taxon were corrected based on the consumer's trophic position (TP) and the respective isotope enrichment factors. The TP of each consumer taxon was estimated based on the difference in the δ^15^N values of consumers and the trophic baseline organisms, and the isotope enrichment factor per trophic level (Post 2002):
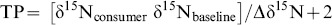
(2)where δ^15^N_consumer_ is the average δ^15^N of the consumer whose TP is estimated here; δ^15^N_baseline_ is the average δ^15^N of the dietary organism. We used the average δ^15^N value of zooplankton (the water column phytoplankton feeder) and the stream crab (the benthic grazer) as trophic baseline organisms. The Δδ^15^N is the isotope fractionation level during assimilation of dietary nitrogen by the animal. An average value of 3.4‰ from the literature [37] was used to correct the isotope enrichment per trophic level.

To prepare isotope data for IsoSource model estimates, the consumer's TP above the primary producer (i.e., TP-1) was multiplied by the isotope enrichment factor of 0.4‰ for δ^13^C and 3.4‰ for δ^15^N to obtain the taxon-specific isotope enrichment levels which were then added to each production source. The mean δ^13^C and δ^15^N values for each taxon and the five production sources (corrected for isotope enrichment) were analyzed with the IsoSource mixing model at an increment level of 1% and a tolerance level of 0.1‰.

Selected hydrological and environmental variables for the pre-dam (1999 to 2003) and post-dam (2004–2005) periods (this study period) were collected from the literature and monitoring reports to evaluate the hydro-ecological conditions at locations near the study sites.

Statistical analysis was performed using SigmaPlot software (Version 12.5, Systat Software, Inc. San Jose, CA). Data set was first examined for normality using the Shapiro-Wilk procedure. One-way Analysis of Variance (ANOVA) was used following by pairwise comparison using the Holm-Sidak method. When normality test failed, non-parametric analysis (Kruskal-Wallis ANOVA on Ranks) was used followed by Duncan's multiple comparison. Two-way ANOVA was used to examine the interactive effects of study sites and stable isotopes. Statistical difference was considered to be significant at p<0.05.

## Results

### Environmental and isotopic variations

Selected eco-hydrological variables measured before (1999–2003) and after the TGD construction (2004–2005) were compared to evaluate the impacts of damming to hydrological regime and the water quality near the three study sites (Table 1). Flow rates were lower at all sites following dam construction. Interannual variation in rainfall may have contributed to the observed pattern of flow reduction before and after dam construction. Current studies indicate that the hydrological regime at the Luoqi, which is 500 Km upper stream of the TGD, was not impacted. However, the flow rates at the two downstream sites decreased by about 90% compared to the pre-dam period. The hydrological regime at Huanghua has changed from a typical constricted channel to an estuarine habitat since the TGD started storing water in 2003.The flow rate (the average from the wet and dry seasons) at Maoping were 0.01 and 0.03 m s^−1^ in 2004 and 2005, respectively. High nutrient concentrations have been found at all study sites before and after TGD construction. Primary productivity as Chl a concentration remained low at Luoqi and was considerably elevated at the two downstream sites, especially at Maoping (Table 1), likely as the results of the decreased flow rate and improved water clarity.

**Table 1:** Averages of selected environmental variables determined before (1999–2003) and after (2004–2005) the Three Gorge Dam construction at river sections near the three study sites along Yangtze River.

Variable	Upstream		Midstream		Downstream	
	Before	After	Before	After	Before	After
Flow rate, m s^−1^	3.20	1.75	1.29	0.36	1.45	0.17
Chl a, mg m^−3^	4.1	2.9	10.2	14.7	5.2	15
TP, mg L^−1^	0.194	0.216	0.105	0.145	0.140	0.138
TN, mg L^−1^	2.005	1.665	1.559	1.494	1.260	1.357
pH, SU	7.9	7.3	7.9	7.9	8.2	8.2
DO, mg L^−1^	7.63	7.28	8.39	8.04	8.11	8.97
Conductivity, µs cm^−1^	232	248	267	372	268	279
COD, mg L^−1^	12.95	13.64	10.12	10.95	11.18	16.18
BOD, mg L^−1^	2.29	1.68	2.07	1.13	1.43	1.77

Data are taken from various reports [30], [52], [55], [56].

Stable isotope compositions of terrestrial organic matter, C_3_ and C_4_ plants collected from different sites are shown in Fig. 3 and Table S1. The average δ^13^C values of the terrestrial plants spanned a narrow range, from −29.8 to −28.6‰ for C_3_ plants and −13.5 to −12.2‰ for C_4_ plants. The average δ^15^N values of these plants were more variable within study sites, ranging from 0.2 to 2.3‰ for C_3_ plants and from −4.3 to 2.1‰ for C_4_ plants. Among the algal taxa, the average isotope compositions of FPOM varied slightly from −25.5 to −23.8‰ for δ^13^C and from 3.6 to 4.6‰ for δ^15^N among sites and were more depleted at Huanghua than other sites (Fig. 3). Epiphytic algae displayed the highest δ^13^C values (−20.2 to −18.3‰) and moderate δ^15^N values (4.9 to 6.8‰) among sites. By contrast, the FA displayed moderate δ^13^C values (−23.7 to −21.7‰) and the highest δ^15^N values (9.4 to 11.1‰). There was no consistent trend of isotope changes from the upstream to the downstream site (Fig. 3).

Consumer δ^13^C varied from −24.9 to −17.1‰ at Luoqi, −24.0 to −20.5‰ at Huanghua, and −23.6 to −17.1‰ at Maoping (Fig. 3, and Table S2, S3 and S4). There were no significant differences in consumer δ^13^C between the dry and wet period within each site (Two-tail paired T test, all p≥0.05). Pooled data from the dry and wet periods from each site also showed no significant differences among sites (Kruskal-Wallis Analysis, H = 1.912, df = 2, p = 0.38). Consumer δ^15^N varied from 5.6 to 12.1‰ at Luoqi, 5.2 to 13.5‰ at Huanghua, and 5.1 to 14.9‰ at Maoping (Table S2, S3 and S4). There was no significant difference in consumer δ^15^N at Luoqi between the dry and wet period (Two-tail paired T test, p = 0.59). However, there were significant differences in consumer δ^15^N at Huanghua (Two-tail paired T test, p = 0.001) and Maoping (Two-tail paired T test, p = 0.001) between the dry and wet period. During the dry period, the average consumer δ^15^N increased from Luoqi (8.4‰), Huanghua (9.8‰) to Maoping (10.1‰) and displayed significant differences among sites (ANOVA, F = 8.1, p = 0.003) although the midstream site (Huanghu) and downstream site (Maoping) did not differ significantly (Duncan's method, p = 0.47). However, there were no significant differences in consumer δ^15^N among sites during the wet period (ANOVA, F = 0.48, p = 0.62).

### Carbon sources supporting river consumers

Consumer δ^13^C at Luoqi had a range of 8.9‰ which is smaller than the δ^13^C range of the five production sources (Fig. 3). The IsoSource model estimates (1^st^ and 99^th^ percentile) indicated that terrestrial plants were the dominant carbon sources to consumer production (Table 2). The average 1^st^ and 99^th^ percentile contributions from C_3_ and C_4_ plants to the 10 consumer taxa ranged from 3 to 43% and 20 to 33%, respectively. The C_3_ plants supported between 17–61% and 12–58% of the production of zooplankton and the Chinese minnow while contributions to the other taxa were less significant. The contribution from C_4_ plants to each consumer taxon was similar with a few exceptions. The 1^st^ and 99^th^ percentile values were typically over 20 and less than 40%, respectively for the majority of consumer taxa. Zooplankton, Chinese minnows and Chinese perch received less than 20% from C_4_ plants. However, the stream crab received between 40 and 59% of its carbon from C_4_ plants. The 1^st^ percentile values of algal carbon for each consumer taxon were zero although the 99^th^ percentile values of FPOM were typically greater than 60%. The FA displayed the lowest 1^st^ and 99^th^ percentile values (Table 2).

**Table 2:** 1st and 99th percentiles of the contribution of the five production sources to the 10 consumers taxa in the upstream (Luoqi) of the Three Gorges Dam.

Taxa	C_3_ plants	C_4_ plants	FPOM	EA	FA
Zooplankton	41–67	19–26	0–39	0–13	0–7
Stream Crab	0–5	39–55	0–8	13–55	0–31
Mixed snails	<0.1–43	20–32	0–70	0–32	0–24
River shrimp	0–41	21–34	0–66	0–33	0–25
Common carp	0–35	24–38	0–57	0–36	0–27
Crucian carp	0–41	21–34	0–67	0–33	0–25
Bronze gudgeon	<0.1–47	18–30	0–75	0–30	0–23
Yellow catfish	<0.1–45	19–31	0–73	0–31	0–23
Chinese minnow	15–58	13–23	0–70	0–25	0–19
Chinese perch	<0.1–47	18–30	0–76	0–30	0–22

Note: Percentiles were estimated using a five-source dual isotope mixing model in the IsoSource program (Philips and Gregg 2003). FPOM: Fine particulate organic matter; EA: Epiphytic algae; FA: Filamentous algae.

The consumer taxa at Huanghua had a considerably narrower δ^13^C range (3.2‰) than at Luoqi (Fig. 3). Unlike the upstream site where C_3_ plants showed sporadic support to consumer production, the majority of the consumers at Huanghua were supported by both C_3_ and C_4_ plants. This was especially consistent for the C_3_ plants as the 1^st^ and 99^th^ percentile values were all high with a typical range between 40 and 50% (Table 3). On average, C_3_ plants contributed between 47 and 57% of the consumer production at Huanghua while the average contribution from C_4_ plants fell between 28 and 36%. The Chinese perch, which is a piscivore, received the highest support (57 to 68%) from C_3_ plants through trophic transfers. Similar to the upstream site, C_4_ plants contributed the most to stream crab production with a range of 36 to 43%. Also similar to the upstream site, the model results revealed that all algal sources had a 1^st^ percentile value of zero. One noticeable difference was the 99^th^ percentile value of FPOM (typically below 20%) at Huanghua that was considerably lower than those at the upstream site.

**Table 3:** 1st and 99th percentiles of the contribution of the five production sources to the ten consumer taxa at the midstream (Huanghua) site of the Three Gorges Dam.

Taxa	C_3_ plants	C_4_ plants	FPOM	EA	FA
Zooplankton	46–59	23–32	0–25	0–22	0–7
Stream crab	38–50	34–42	0–23	0–20	0–7
Mixed snails	49–63	19–28	0–24	0–23	0–8
River shrimp	39–51	33–41	0–22	0–20	0–7
Common carp	44–57	26–35	0–20	0–21	0–7
Crucian carp	41–54	30–38	0–23	0–21	0–7
Bronze gudgeon	42–54	29–37	0–24	0–21	0–7
Yellow catfish	41–54	30–38	0–23	0–21	0–7
Chinese minnow	46–59	23–32	0–25	0–22	0–7
Chinese perch	53–67	13–23	0–27	0–24	0–8

Note: Same as in Table 2.

The δ^13^C values of the consumer assemblage collected in the limnetic zone (Maoping) of the TGR had a range of 4.8‰ while the production sources had a range of 16.6‰ (Fig. 3). Model estimates showed that both C_3_ and C_4_ plants were the dominant contributors to most of the consumer taxa (Table 4). The average 1^st^ and 99^th^ percentile values to the consumer taxa were 17–42% for C_3_ and 17–32% for C_4_ plants which were considerably lower than those at Huanghua. Zooplankton, the bronze gudgeon, the yellow catfish and the Chinese perch all depended more upon C_3_ organic matter, directly or indirectly, than the other taxa (Table 4). Again, the stream crab utilized more organic matter from C_4_ (34 to 51%) than C_3_ plants (0 to 15%). However, other taxa showed similar percentile values for the contribution from C_4_ plants (Table 4). Unlike at Luoqi and Huanghua where all algal sources had a 1^st^ percentile value of zero, the model estimates revealed some usage of algal carbon at Maoping. In addition to terrestrial plants, the crab also relied on planktonic carbon (1 to 38%) while zooplankton had 1^st^ and 99^th^ percentile values of 2 to 31% for the epiphytic algae. However, other consumer taxa showed no signs of a significant use of algal carbon although the 99^th^ percentile value for FA and FPOM at Maoping increased significantly or remained as high as those in other sites.

**Table 4:** 1st and 99th percentiles of the contribution of the five production sources to the ten consumer taxa at the limnetic zone (Maoping) site of the Three Gorges Dam.

Taxa	C_3_ plants	C_4_ plants	FPOM	EA	FA
Zooplankton	22–50	12–26	0–61	0–36	0–22
Stream crab	0–16	33–51	0–37	0–48	0–30
Mixed snails	12–42	17–32	0–66	0–38	0–24
River shrimp	16–45	15–30	0–64	0–37	0–23
Common carp	12–42	17–32	0–66	0–38	0–24
Crucian carp	15–42	17–32	0–66	0–38	0–24
Bronze gudgeon	23–50	12–26	0–61	0–35	0–22
Yellow catfish	23–50	12–25	0–61	0–35	0–22
Chinese minnow	6–37	20–35	0–69	0–40	0–25
Chinese perch	21–48	13–27	0–62	0–36	0–22

Note: Same as in Table 2.

## Discussion

### The dominance of terrestrial carbon sources

The IsoSource mixing model identified terrestrial organic matter, both C_3_ and C_4_ plants, as the dominant carbon source supporting consumer production at the three study sites upstream of the TGD (Table 5). Our results support other studies which demonstrated the essential role of terrestrial organic matter for aquatic production in lakes [5], [8], streams [12], [38], [48] and rivers [12], [23], [25], [57]. However, our results conflict with studies suggesting that the terrestrial source is not important to the aquatic food web [6], [10], [16], [22], [46]. We argue that in large rivers with high turbidity, high flow and greater depth such as in the Yangtze River, planktonic and benthic algal production is severely limited. The production of aquatic macrophytes in the Yangtze River is also limited as the result of a steep slope, a hard river bottom and little littoral zone area for the development of macrophytes. These impacts result in an insufficient supply of autochthonous production to meet the growth demand of the river consumers. Consequently, terrestrial loading of organic matter becomes an important or a dominant carbon source to aquatic consumer production. The relative importance of terrestrial and algal carbon to the aquatic food web is highly variable among rivers where the geological location, geometry, hydrological pattern, system productivity, floodplain size and land use characteristics differ greatly. These factors may explain the contrasting findings in the literature [11], [57].

**Table 5:** Averages of 1st and 99th percentile values of each production source contribution to the 10 consumer taxa in the three study sites calculated from the IsoSource program.

Site	C_3_ plants	C_4_ plants	FPOM	EA	FA
Luoqi	6–43	21–33	0–60	1–32	0–21
Huanghua	44–57	26–35	0–24	0–22	0–7
Maoping	15–42	17–32	0–61	0–38	0–24

Note: Same as in Table 2.

While the importance of terrestrial C_3_ plants to the river food web has been demonstrated in a number of studies (although conflicting findings exist), our model results also pointed to the significant contribution of terrestrial C_4_ grasses to consumer production at all three upstream sites of the TGD (Table 5). Our results conflict with other studies that suggest that C_4_ plants contributed little to the river food web [46], [57]. However, a few additional studies showed that the C_4_ contribution to the river food web was more significant during the wet season, as in-stream primary production is hampered by high flow and high turbidity [24], [29], [49]. Model estimates indicate that C_3_ and C_4_ carbon were assimilated simultaneously by primary consumers or other consumers at higher trophic positions through the food chains, suggesting that both C_3_ and C_4_ plants play a comparatively large role as a dietary source to river consumers. Another transfer mechanism of C_4_ carbon to aquatic food webs is the consumption of terrestrial invertebrates which feed on C_4_ plants by fish of higher trophic positions. Several studies indicated that transfer of invertebrates-derived nutrients from land can be a significant contribution to aquatic ecosystem [1], [27], [39]. Given the mixed results from different studies, it is likely that C_4_ grasses, the availability of other resources, consumer composition and habitat heterogeneity may influence the contribution of C_4_ plants to the consumer production in different rivers.

The sum of the 99^th^ percentile values from C_3_ and C_4_ plants at a given site cannot account for 100% of the production sources to consumer production (Table 5). This implies that autochthonous production also contributed to the local food web. The low 99^th^ percentile value of the filamentous algae ruled out the possibility of it having a supplementary role in consumer production. However, the FPOM and EA may have served as contributors to consumer production in the Yangtze River. This is supported by the high 99^th^ percentile values of FPOM and EA, especially at Luoqi and Maoping. Model estimates showed non-zero values for the 1^st^ percentile associated with FPOM and EA in two invertebrates from the downstream site. This may be an indication of increasing algal production as the consequence of reduced flow near the TGD. However, the contribution from algal carbon was low and was limited to a few taxa, suggesting an insignificant role of autochthonous production in supporting the aquatic food web in the middle Yangtze River. This is at least the case during our study period less than a year after TGD construction.

### Tests of river conceptual models

The river continuum concept (RCC) hypothesizes that large rivers receive the majority of the organic matter, supporting their food webs, from terrestrial loading to headwater and mid-order streams [47]. The flood pulse concept (FPC) proposes that river food webs are more dependent on production derived from the floodplain than upon organic matter transported from tributaries upstream [26]. The FPC includes aquatic macrophytes as an important input but algal carbon is less important. We do not have direct evidence to evaluate the importance of organic carbon from the head waters, but given the length of Yangtze River and the presence of numerous tributaries along the main river channel, it is unlikely that the carbon source described in the RCC model provides the main energy flow to river consumers in the middle Yangtze River.

The results of the current study support our hypothesis that suggests the importance of terrestrial carbon for river consumers at all three hydrologically different reaches of the middle Yangtze River. We also found that the contribution from C_4_ carbon is as important as that from C_3_ plants in the current study. Some studies suggest that terrestrial carbon, due to its low nutrient value, is supplemental at best to the primary consumers. We offer three explanations to account for the consumer dependence of terrestrial carbon in the Yangtze River and other large rivers. First, algal production in the Yangtze River, as well as in some other large rivers, is not sufficient to support the secondary production. This is due to several well-recognized constraints (high turbidity, high flow rate and a deep water column) which severely limit algal production. For example, the pristine site (Luoqi) upstream of the TGD had low Chl a concentrations before and after dam construction despite high nutrient concentrations (Table 1). Wu et al [51] reported the results of a stable isotope study on the sources of carbon within the Yangtze River system from May 1997 and May 2003. They found that the δ^13^C values of the particulate organic carbon (POC) pool were consistent with the isotope signature of the terrestrial soil organic carbon in the Yangtze River and that the planktonic algal carbon was a minor component of the POC pool. Second, plant detritus from the floodplain, unlike that from the headwater, can be of high nutritional value. For example, some agriculture crops (legume species) had a C/N ratio as low as that of the algal biomass (Table S1). On the other hand, the nutritional value of terrestrial detritus can be improved by microbial colonization upon entering the aquatic ecosystems [2], [32]. In addition, some plant parts, such as seeds and fruits, which are apparently high in nutritional value, may be directly consumed by tropical aquatic animal species [19]. Finally, terrestrial organic matter can enter the river food web through the consumption of terrestrial invertebrates by fish, as discussed above. In conclusion, environmental constraints of autochthonous production and an ample supply of terrestrial organic matter, with relatively high nutrient content, can lead to terrestrial carbon sources dominating the aquatic production in many large rivers.

The riverine-productivity model [44] emphasizes the importance of algal carbon to river consumers. Support of aquatic consumers by algal carbon has been well documented in streams and rivers. The existence of evidence for supporting both sides (the RPM and FPC) in the literature, strongly suggests that given the high variability of hydrology, geomorphology and taxonomy of large rivers, a single riverine source model is not sufficient to predict the carbon sources of the world's rivers [57]. In some cases, both models are needed to account for the energy flow to the food webs of large rivers. Habitat heterogeneity, fragmentation and localized eutrophication may lead to the dominance of different sources of organic matter, either by terrestrial contributions or by algal production for consumer production within rivers [24], [57]. This is especially true for large rivers whose drainage basins have experienced intensive land-use development and hydro-dam construction.

### Damming effects

Damming is the single most important factor influencing river connectivity and its ecological functions [14], [15], [42]. This study was conducted at one and one and a half years after the TGD was completed. The effects of damming to the upstream hydrology and water quality were almost immediate (Table 1). Changes in fish species composition after dam construction also occurred as the numbers of species in Yangtze River decreased greatly [53]. This is also evidenced by the decrease in the number of fish species collected at the three study sites (Table S2, S3 and S4). However, significant increases in phytoplankton production at Maoping (Chl a, Table 1) was not well reflected in the isotope composition of the consumers. The importance of surface water algal blooms at Huanghua and especially at Maoping to the consumers may have been reduced as a consequence of a rapid decrease in light penetration and the greater depth of the water column (80 m) in the TGR. Under these circumstances, the amount of algal carbon available to the consumer taxa appears to be much less significant compared to the contribution of terrestrial carbon which is expected to increase at the limnetic zone of TGR. In fact, model estimates showed that limited amount of algal carbon to the consumer production at Maoping was present in a few invertebrate taxa. This is contrary to our second hypothesis which suggested that the planktonic algal contribution would dominate the food web in the river reaches affected by the TGD construction. Since our study was conducted shortly after the completion of TGD, further study is needed to examine if the major carbon sources to the consumer food web have changed at a longer time scale after TGD completion.

### Constraints of stable isotope analysis and model estimates

Our model estimates based on data from three upstream reaches of the TGD for two hydrological events provide some temporally and spatially integrated information on the importance of terrestrial carbon to the river food web. At each site, the isotope values of the terrestrial source were relatively consistent between the dates while the isotope values of the autochthonous production in rivers will likely change seasonally [28]. Therefore, the isotope signatures of the algal samples collected in May and September may only provide a brief snapshot of the two hydrologically contrasting periods. Future studies should consider sampling at an increased frequency to better capture the temporal variations in stable isotope values and thereby provide more accurate model estimates. In addition, our estimates of planktonic algal contributions might be affected by the appropriateness of FPOM as an algal surrogate [20]. The use of an algal concentration technique such as colloidal silica centrifugation [10] can provide better isotope signals for the algae. Recent research has revealed large differences in hydrogen stable isotope values for terrestrial and aquatic production, which are also reflected in the aquatic consumers [8], [12]. This technique could be used to further elucidate the relative contribution of terrestrial and aquatic carbon to the various consumers in large rivers.

Understanding energy flow and nutrient cycling pathways in the food web of large rivers is essential in the planning for wildlife conservation and environmental protection [14], [18], [34], [51]. Our study using stable isotope analysis and the IsoSource model estimates revealed the important roles of terrestrial carbon to consumer production in the middle Yangtze River. With rapid urbanization in China and other developing countries, protection of riparian integrity and stability is a prerequisite for better water resource management. Our data may also serve as baseline information on the initial effects of the construction of the TGD, the world largest hydro-dam, which could help direct floodplain management of large rivers around the world.

## Supporting Information

Table S1 **Average δ^13^C, δ^15^N and C:N ratios for the production sources at the three study sites in the Three-Gorges Reservoir area during the wet and dry periods between 2004 and 2005.**
(DOCX)

Table S2 **Average, standard deviation (SD) of δ^13^C, δ^15^N ratios for all consumer taxa at the upstream site (Luoqi) in Three-Gorges Reservoir during the wet and dry periods between 2004 and 2005.**
(DOCX)

Table S3 **Average, standard deviation (SD) of δ^13^C, δ^15^N ratios for all consumer taxa at the midstream site (Huanghua) in Three-Gorges Reservoir during the wet and dry periods between 2004 and 2005.**
(DOCX)

Table S4 **Average, standard deviation (SD) of δ^13^C, δ^15^N ratios for all consumer taxa at the upstream site (Maoping) in Three-Gorges Reservoir during the wet and dry periods between 2004 and 2005.**
(DOCX)
