# Supplementary material for: Terrestrial Contributions to the Aquatic Food Web in the Middle Yangtze River
Source: PLoS One. 2014 Jul 21;9(7):e102473. doi: 10.1371/journal.pone.0102473 (PMC4105416; doi:10.1371/journal.pone.0102473)
Supplement: Table S1 — Average δ13C, δ15N and C:N ratios for the production sources at the three study sites in the Three-Gorges Reservoir area during the wet and dry periods between 2004 and 2005. (DOCX) [file pone.0102473.s001.docx]

| Table S1. Average δ^13^C, δ^15^N and C:N ratios for the production sources at the three study sites in Three-Gorges Reservoir during the wet and dry periods between 2004 and 2005. | | | | | | | | |
| --- | --- | --- | --- | --- | --- | --- | --- | --- |
|  | Wet period (September 2004) | | | | Dry period (May 2005) | | | |
|  | δ^13^C | δ^15^N | C:N | n | δ^13^C | δ^15^N | C:N | n |
| Luoqi |  |  |  |  |  |  |  |  |
| C3 | -29.5(1.0) | 2.0(3.3) | 13.4(4.5) | 24 | -28.6(1.1) | 1.7(3.4) | 12.8(3.8) | 21 |
| C4 | -13.5(1.1) | -4.3(2.7) | 26.3(5.2) | 12 | -13.0(0.6) | -2.7(1.3) | 23.1(2.9) | 6 |
| EA | -17.9(0.4) | 6.3(1.2) | 6.5(0.3) | 5 | -19.2(0.5) | 7.0(0.9) | 5.3(0.2) | 5 |
| FA | -20.6(0.4) | 9.9(0.3) | 5.9(0.7) | 5 | -24.6(0.7) | 11.6(0.7) | 5.5(0.3) | 3 |
| CPOM | -22.5(1.7) | 0.7(1.2) | 17.6(0.9) | 5 | -26.3(0.7) | 2.6(1.0) | 16.6(1.3) | 5 |
| FPOM | -26.2(1.1) | 2.7(1.9) | 13.1(0.7) | 5 | -24.2(1.0) | 4.1(0.7) | 9.3(0.5) | 3 |
| Huanghua |  |  |  |  |  |  |  |  |
| C3 | -29.8(0.7) | 0.2(2.0) | 15.7(5.6) | 26 | -29.3(1.0) | 1.3(2.7) | 14.8(5.1) | 24 |
| C4 | -12.2(1.1) | 1.3(1.5) | 20.3(4.1) | 9 | -12.6(0.6) | 2.1(1.4) | 21.4(4.8) | 9 |
| EA | -18.8(0.7) | 4.2(2.0) | 6.1(0.4) | 5 | -20.9(0.5) | 5.2(1.1) | 6.7(0.3) | 5 |
| FA | -21.0(1.6) | 11.2(0.8) | 6.0(0.3) | 5 | -22.0(0.9) | 11.1(0.5) | 7.2(0.3) | 5 |
| CPOM | -23.6(1.3) | 0.4(1.4) | 18.3(1.3) | 5 | -25.9(0.7) | 1.9(0.5) | 16.0(1.0) | 5 |
| FPOM | -25.5(1.2) | 2.6(0.7) | 12.6(0.4) | 3 | -23.0(0.7) | 5.3(1.0) | 9.6(0.7) | 5 |
| Maoping |  |  |  |  |  |  |  |  |
| C3 | -29.7(1.2) | 2.3(1.9) | 13.3(3.7) | 12 | -29.6(1.2) | 2.3(2.5) | 14.1(3.4) | 12 |
| C4 | -12.8(0.5) | -0.2(1.4) | 19.7(3.1) | 7 | -13.1(0.8) | 0.3(1.6) | 17.9(2.9) | 6 |
| EA | -18.5(1.4) | 6.2(1.3) | 6.7(0.3) | 5 | -20.3(0.7) | 5.7(2.0) | 5.6(0.4) | 5 |
| FA | -23.4(1.0) | 9.3(0.7) | 6.8(1.3) | 5 | -23.9(0.3) | 9.5(0.5) | 7.4(0.5) | 3 |
| CPOM | -25.2(0.7) | 1.7(0.6) | 21.1(1.2) | 5 | -24.2(1.2) | 2.5(0.4) | 18.2(0.6) | 3 |
| FPOM | -25.2(1.0) | 3.5(2.8) | 12.1(0.3) | 4 | -23.3(0.6) | 5.1(0.5) | 8.1(0.4) | 3 |
| Note: C3 and C4: C3 and C4 plants; EA: Epiphytic algae; FA: Filamentous algae; FPOM: Fine particulate organic matter; CPOM: Coarse particulate organic matter. | | | | | | | | |
