# Supplementary material for: Terrestrial Contributions to the Aquatic Food Web in the Middle Yangtze River
Source: PLoS One. 2014 Jul 21;9(7):e102473. doi: 10.1371/journal.pone.0102473 (PMC4105416; doi:10.1371/journal.pone.0102473)
Supplement: Table S3 — Average, standard deviation (SD) of δ13C, δ15N ratios for all consumer taxa at the midstream site (Huanghua) in Three-Gorges Reservoir during the wet and dry periods between 2004 and 2005. (DOCX) [file pone.0102473.s003.docx]

| Table S3. Average, standard deviation (SD) of δ^13^C, δ^15^N ratios for all consumer taxa at the midstream site (Huanghua) in Three-Gorges Reservoir during the wet and dry periods between 2004 and 2005. | | | | | | | | | | |
| --- | --- | --- | --- | --- | --- | --- | --- | --- | --- | --- |
| Species | Wet period (September 2004) | | | | | Dry period (May 2005) | | | | |
|  | δ^13^C | SD | δ^15^N | SD | n | δ^13^C | SD | δ^15^N | SD | n |
| Zooplankton | -21.8 | 1.2 | 5.2 | 1.6 | 5 | -23.8 | 0.8 | 6.7 | 0.5 | 5 |
| Mixed snails | -23.2 | 0.5 | 8.5 | 0.6 | 3 | -23.1 | 0.8 | 8.1 | 0.5 | 3 |
| *Macrobranchium nipponense* | -20.8 | 0.4 | 8.2 | 0.8 | 3 | -20.9 | 0.3 | 8.6 | 0.5 | 3 |
| *Sinopotamon yangtsekiense* | -20.5 | 1.1 | 6.3 | 1.0 | 3 | -19.7 | 0.9 | 10.7 | 0.7 | 3 |
| *Ctenopharyngodon idellus* | -23.6 | 0.4 | 6.2 | 0.2 | 3 | -22.9 | 0.4 | 8.1 | 0.3 | 3 |
| *Cyprinus carpio* | -21.4 | 0.6 | 7.5 | 0.6 | 3 | -22.8 | 0.6 | 8.7 | 0.6 | 3 |
| *Carassius auratus* | -21.3 | 0.9 | 9.2 | 0.5 | 3 | -21.3 | 0.8 | 11.6 | 0.4 | 3 |
| *Coreius guichenoti* | -22.3 | 0.4 | 9.3 | 0.4 | 3 | -20.8 | 0.6 | 9.7 | 0.2 | 3 |
| *Hemibarbus maculatus* | -22.9 | 1.1 | 9.4 | 0.2 | 3 | -22.1 | 0.4 | 10.2 | 0.4 | 3 |
| *Pelteobagrus fulvidraco* | -20.1 | 1.6 | 9.8 | 1.7 | 3 | -22.4 | 0.9 | 11.5 | 1.2 | 3 |
| *Distoechodon tumirostris* | -21.8 | 0.1 | 10.6 | 0.2 | 3 | -22.2 | 0.2 | 12.7 | 0.5 | 3 |
| *Silurus asotus* | -21.4 | 0.5 | 11.7 | 1.2 | 3 | -21.5 | 0.8 | 13.7 | 0.7 | 3 |
| *Hemiculterella sauvagei* | -22.0 | 0.6 | 8.7 | 0.3 | 3 | -22.9 | 0.6 | 9.4 | 0.4 | 3 |
| *Siniperca* sp. | -23.1 | 1.1 | 11.9 | 0.7 | 3 | -24.0 | 0.6 | 13.5 | 0.6 | 3 |
| *Hypophthalmichthys molitrix* | -21.3 | 0.9 | 7.2 | 0.4 | 3 | -22.1 | 0.3 | 8.1 | 0.1 | 3 |
| *Megalobrama amblycephala* | -22.9 | 0.4 | 8.2 | 0.3 | 3 | -23.7 | 0.1 | 9.5 | 0.4 | 3 |
| Note: For invertebrates, N is the number of composite samples; See text for more details. | | | | | | | | | | |
