# Supplementary material for: Terrestrial Contributions to the Aquatic Food Web in the Middle Yangtze River
Source: PLoS One. 2014 Jul 21;9(7):e102473. doi: 10.1371/journal.pone.0102473 (PMC4105416; doi:10.1371/journal.pone.0102473)
Supplement: Table S4 — Average, standard deviation (SD) of δ13C, δ15N ratios for all consumer taxa at the upstream site (Maoping) in Three-Gorges Reservoir during the wet and dry periods between 2004 and 2005. (DOCX) [file pone.0102473.s004.docx]

| Table S4. Average, standard deviation (SD) of δ^13^C, δ^15^N ratios for all consumer taxa at the upstream site (Maoping) in Three-Gorges Reservoir during the wet and dry periods between 2004 and 2005. | | | | | | | | | | |
| --- | --- | --- | --- | --- | --- | --- | --- | --- | --- | --- |
| Species | Wet period (September 2004) | | | | | Dry period (May 2005) | | | | |
|  | δ^13^C | SD | δ^15^N | SD | n | δ^13^C | SD | δ^15^N | SD | n |
| Zooplankton | -23.2 | 1.3 | 5.1 | 1.9 | 5 | -23.6 | 0.7 | 7.0 | 2.6 | 5 |
| Mixed snails | -22.2 | 0.6 | 7.2 | 0.6 | 3 | -22.1 | 0.6 | 7.3 | 0.3 | 3 |
| *Macrobranchium nipponense* | -21.8 | 0.5 | 8.6 | 1.0 | 3 | -22.7 | 0.6 | 8.2 | 1.1 | 3 |
| *Sinopotamon yangtsekiense* | -17.1 | 0.5 | 6.4 | 0.9 | 3 | -21.1 | 0.2 | 9.7 | 1.8 | 3 |
| *Ctenopharyngodon idellus* | -22.7 | 0.3 | 7.2 | 0.3 | 3 | -23.4 | 0.4 | 8.7 | 0.5 | 3 |
| *Cyprinus carpio* | -22.4 | 0.5 | 8.9 | 0.8 | 3 | -21.4 | 0.1 | 10.1 | 0.6 | 3 |
| *Carassius auratus* | -23.7 | 1.2 | 9.5 | 0.4 | 3 | -22.0 | 1.0 | 10.7 | 0.5 | 3 |
| *Coreius guichenoti* | -22.5 | 0.5 | 9.6 | 0.6 | 3 | -23.4 | 0.4 | 11.3 | 0.4 | 3 |
| *Pelteobagrus fulvidraco* | -24.4 | 1.0 | 11.4 | 1.0 | 3 | -23.0 | 0.8 | 14.6 | 1.1 | 3 |
| *Silurus asotus* | -22.2 | 1.7 | 11.3 | 1.3 | 3 | -22.2 | 0.2 | 13.9 | 0.5 | 3 |
| *Hemiculterella sauvagei* | -21.1 | 0.8 | 7.6 | 0.7 | 3 | -21.7 | 0.3 | 8.0 | 0.3 | 3 |
| *Ophiocephalus argus* | -22.5 | 0.6 | 11.2 | 0.4 | 3 | -23.9 | 1.4 | 13.8 | 0.7 | 3 |
| *Siniperca* sp. | -22.1 | 0.3 | 12.1 | 0.6 | 3 | -22.6 | 0.3 | 14.9 | 0.4 | 3 |
| *Hypophthalmichthys molitrix* | -22.7 | 0.8 | 6.6 | 0.5 | 3 | -23.0 | 0.4 | 8.6 | 0.0 | 3 |
| *Culter erythropterus* | -21.9 | 0.4 | 11.7 | 0.5 | 3 | -22.1 | 0.4 | 13.1 | 0.3 | 3 |
| *Megalobrama amblycephala* | -20.9 | 0.2 | 5.1 | 0.5 | 3 | -22.2 | 0.4 | 8.1 | 0.4 | 3 |
| *Schizothorax prenanti* | -24.0 | 0.6 | 12.4 | 0.7 | 3 | -22.9 | 0.7 | 13.7 | 0.4 | 3 |
| *Leiocassis longirostris* | -21.9 | 0.7 | 12.0 | 0.7 | 3 | -22.4 | 0.4 | 14.1 | 0.3 | 3 |
| Note: For invertebrates, N is the number of composite samples; See text for more details. | | | | | | | | | | |
